# Effect of an information leaflet on breast cancer screening participation: A cluster randomized controlled trial

**DOI:** 10.1186/s12889-021-11360-0

**Published:** 2021-07-03

**Authors:** Jose Maria Montero-Moraga, Margarita Posso, Marta Román, Andrea Burón, Maria Sala, Xavier Castells, Francesc Macià

**Affiliations:** 1 Department of Epidemiology and Evaluation, IMIM (Hospital del Mar Medical Research Institute), Passeig Marítim 25-29, 08003 Barcelona, Spain; 2 Preventive Medicine and Public Health Training Unit PSMar-ASPB-UPF, Passeig Marítim 25-29, 08003 Barcelona, Spain; 3 Research Network on Health Services in Chronic Diseases (REDISSEC), Barcelona, Spain

**Keywords:** Early detection of Cancer, Diagnostic screening programs, Breast neoplasms, Information leaflet, Screening participation, Screening attendance

## Abstract

**Objective:**

To evaluate the impact of an information leaflet about the risk-benefit balance of breast cancer screening on women’s participation.

**Methods:**

This cluster randomized controlled trial was conducted within a population-based breast cancer screening program and included women from the catchment areas of two hospitals in Barcelona, Spain. We evaluated women aged 50–69 years invited to screening between September 2019 and January 2020. The intervention group received an information leaflet on the benefits and harms of mammography screening. The control group received the usual invitation letter. The clusters consisted of the processing days of the invitation letter, assigned to the intervention with a simple random allocation scheme. We compared the participation rate at the individual level between groups, stratified by hospital and by per-protocol and intention-to-treat analyses.

**Results:**

We included 11,119 women (137 clusters): 5416 in the intervention group (66 clusters) and 5703 in the control group (71 clusters). A total of 36% (1964/5393) of the women in the intervention group and 37% (2135/5694) of those in the control group attended screening, respectively. Overall, we found no differences in participation among groups (difference in participation − 1.1%; 95%CI; − 2.9 to 0.7%). In a hospital attending a population with a low socioeconomic status, attendance was lower in the intervention group (− 1.4, 95%CI: − 5.7% to − 0.03%).

**Conclusions:**

Overall participation in our program was unaffected by a new information leaflet on the risk-benefit balance of breast cancer screening. However, participation was lower in certain populations with lower socioeconomic status

**Trial registration:**

Trial registration number ISRCTN13848929.

**Supplementary Information:**

The online version contains supplementary material available at 10.1186/s12889-021-11360-0.

## Highlights

- An information leaflet on the benefits and risks of breast cancer screening programs did not affect overall participation.
- The information leaflet decreased participation in an area with low socioeconomic status.
- Screening programs sending material with detailed information on the risk-benefit balance of breast cancer screening should consider that participation could decrease in certain areas.

## Background

Breast cancer screening programs reduce breast cancer mortality, although screening is also associated with some harms, such as false positives, overdiagnosis, anxiety and pain [1–3]. The risk-benefit balance of breast cancer screening programs has been extensively discussed [4]. Several reviews have assessed this controversy, gathering and evaluating available studies [2, 5, 6]. Overall, these reviews have concluded that there is evidence of the benefits of these programs, but there is still some uncertainty regarding the magnitude of both the benefits and adverse effects [2, 5, 6]. Thus, governments and institutions have decided to continue offering breast cancer screening, updating the recommendations on which diagnostic tests to use, the periodicity of screening, and the starting and ending ages [1, 7]. A fundamental issue that has been highlighted is the need to provide evidence-based information to women invited to participate [5].

Such information must be balanced, explaining both the benefits and adverse effects of participation, as the general population tends to overestimate the benefits and underestimate the adverse effects of breast cancer screening [8–10]. This information can be presented in different ways, such as interviews with healthcare workers, decision aids, or information leaflets. Few studies have assessed the effect of materials providing information on the benefits and adverse effects of screening programs on participation. Among published studies, most have measured intention to participate, and few have studied effects on screening participation [11–14]. These studies have found that decision aids increase women’s knowledge of breast cancer screening and help them to make an informed choice. However, their results are contradictory: some report a reduction in women’s intention to be screened while others do not, with variability in other outcomes, such as decisional conflict and confidence in the decision [11–14].

In Catalonia, Spain, a new evidence-based leaflet was designed by an expert panel of the Oncology Master Plan of Catalonia and began to be distributed at the end of 2019. The leaflet explains the breast screening program and explicitly addresses the balance between the benefits and harms of breast cancer screening. It provides the number of deaths avoided for participants and the absolute risk of overdiagnosis and explains other possible harms. To our knowledge, it is not known whether the leaflet has had any effect on women’s attendance. Therefore, the aim of this study was to evaluate the impact on participation of this new leaflet targeting women invited to a breast cancer screening program in Barcelona, Spain.

## Methods

### Trial design

We performed a single-centre, cluster randomized, parallel controlled trial, within a breast cancer screening program in Barcelona. The program targets women aged 50 to 69 years, offering them a mammogram every 2 years. The study was carried out in the technical office of Parc de Salut Mar (PSMAR) in Barcelona. This office opened in 1996, with a target population of about 70,000 women and an observed participation rate of around 55% during the last decade. The trial was retrospectively registered as ISRCTN13848929 on 22/04/2021.

### Participants

We included six of the 25 catchment areas of Barcelona covered by the PSMAR, those that sent invitations during the study period. The PSMAR consists of two hospitals: Hospital del Mar (Hospital A), which covers four of the six catchment areas included, and Hospital de l’Esperança (Hospital B), which covers the other two. The six catchment areas cover a target population of 15,825 women per screening round. The 6 catchment areas were chosen because they were pre-scheduled to carry out the screening rounds during the study period. In the city of Barcelona, the breast cancer screening program is organized by areas and screening rounds. In a screening round, all eligible women from one area of the city are invited to breast cancer screening, and when that area is finished, screening invitations are sent to the next area (it is possible that the screening program is active in several areas at the same time). The target population of Hospital A has low socioeconomic status while that of Hospital de l’Esperança has high socioeconomic status. These two populations differ from each other and from the city average regarding socioeconomic status. We included women invited to participate between 30th September 2019 and 17th January 2020, including women who were invited for the first time to the screening program (6746 invited to initial screening). The screening round was defined as a cycle in which a population is invited to screening, indicating how many times screenings have already been offered. Two catchment areas were in the 11th screening round, three were in the 12th round, and the last one was in the 13th round. To identify the women to be invited to screening, the program uses the Catalan registry of persons covered by the publicly-funded health service, which has information on women’s age and residence. We excluded women who had moved residence outside the PSMAR catchment area, as well as census errors. We retrieved the data from the database used by the PSMAR for the daily management of the breast cancer screening program. The database already registers all the information needed for the study, and consequently no informed consent was required from women invited to participate in the program.

### Control condition

Following the usual screening program protocol, we sent invitations to participate in the screening program by surface mail. The control group received the usual letter of invitation to participate in the breast cancer screening program, which includes a personalized letter of invitation with a scheduled date for the mammogram. This date is scheduled three to 4 weeks after the invitation is sent.

### Intervention

The intervention was at the individual level and consisted of adding an information leaflet to the letter. This leaflet, signed by the Catalonian Health Department, describes the breast cancer screening program, and how it is implemented in the region. It explains that in most cases the screening program is beneficial but that it also has risks, and therefore women make the final decision on participation. The leaflet contains qualitative and quantitative information on breast cancer, mortality reduction due to mammography screening, the possibility and advantages of detecting early-stage cancer in participants, and explains how the mammogram is performed (Supplemental file 1). The leaflet also gives the absolute number of breast cancers diagnosed in the region and the number of women who die as a consequence. It also provides the number of women who will need to undergo additional diagnostic tests per 1000 women who participate, the number of cancer diagnoses among them and how many deaths are avoided per 1000 women who regularly attend screening every 2 years from the ages of 50 to 69 years. The leaflet also provides information on the potential risks of screening, explains the meaning of overdiagnosis and overtreatment, and false positives and false negatives. Furthermore, it provides numerical estimates of overdiagnosis, expressed as absolute risk per 1000 participants.

### Randomization

We defined clusters as the processing day of the invitation letter, which were set to be Tuesdays and Fridays. In the usual operation of the screening program, letters to be sent on Mondays and Tuesdays are processed on Friday of the preceding week, and letters to be sent from Wednesday to Friday are processed on Tuesday. We randomized at the cluster level (day of the invitation letter) because it fitted the established operation of the technical screening office and because assignment to days of the invitation letter would not bias randomization. The populations in the different processing days are expected to be similar. We used the RANDOM excel function to perform blind random assignment. The randomization was performed by the research team and the subsequent assignment to groups was done by the screening program staff. The staff also reviewed the women’s residence to exclude women who moved or census errors.

### Outcome

The outcome was participation in the program at the individual level, which is automatically registered in an application at the moment of screening mammography. The follow-up period was initially set to be 90 days, but due to the COVID-19 pandemic we had to deviate from the protocol and shorten it to 30 days. The follow-up period ended on 29th February in Hospital A and on 8th March in Hospital B. The dates differed because there is a two-week interval in Hospital A between the processing date of the letter and the date of the scheduled mammogram, while this period is 3 weeks in Hospital B. This was due to the lower number of invitation letters sent in Hospital B. Women were considered to participate if they were screened during the follow-up period.

### Other variables

Other variables included in the study were place of birth and study level, because in our screening program, women have been found to differ in participation because of these variables. Another variable was initial or subsequent screening, women classified as initial screening had never participated in breast screening while women classified as subsequent screening had participated at least once.

### Sample size

Assuming a participation rate of 55%, we calculated that 4820 letters of invitation were needed for the intervention group and 4820 for the control group to detect a statistically significant difference of 3% in participation between groups. We assumed equal cluster sizes across the processing days of the invitation letter. An alpha risk of 0.05 and a beta risk of 0.2 in a two-sided test was chosen, with an anticipated drop-out rate of 10%. We assumed a participation rate of 55% because it is the observed participation rate in the city of Barcelona.

### Statistical analysis

We obtained participation by dividing the number of women who had a screening mammogram by the number of women invited to participate in the program for each group. Then, we calculated the difference in participation and its 95% confidence interval (95% CI) between the intervention and control groups. Because the socioeconomic status of the covered populations differed between hospitals, we performed an exploratory analysis stratifying by hospital.

We performed an intention-to-treat and a per-protocol analysis. In the per-protocol analysis, we excluded women who reported undergoing mammography in the previous 6 months, as well as those who reported being followed up by another provider. The reason for excluding these women was that they were not considered to be suitable to participate in the screening program, but they could only be identified after we performed the randomization. Since these women were taken from the denominator in the calculation of participation, participation rates were expected to be higher in the per-protocol analyses. Furthermore, there was a higher proportion of women who were followed up by another provider in Hospital B, so excluded women in the per-protocol analyses were mostly from Hospital B. Finally, in an exploratory analysis we compared non-participant and participant characteristics, as well as excluded and included women’s characteristics in the per-protocol analysis.

## Results

**Fig. 1:**
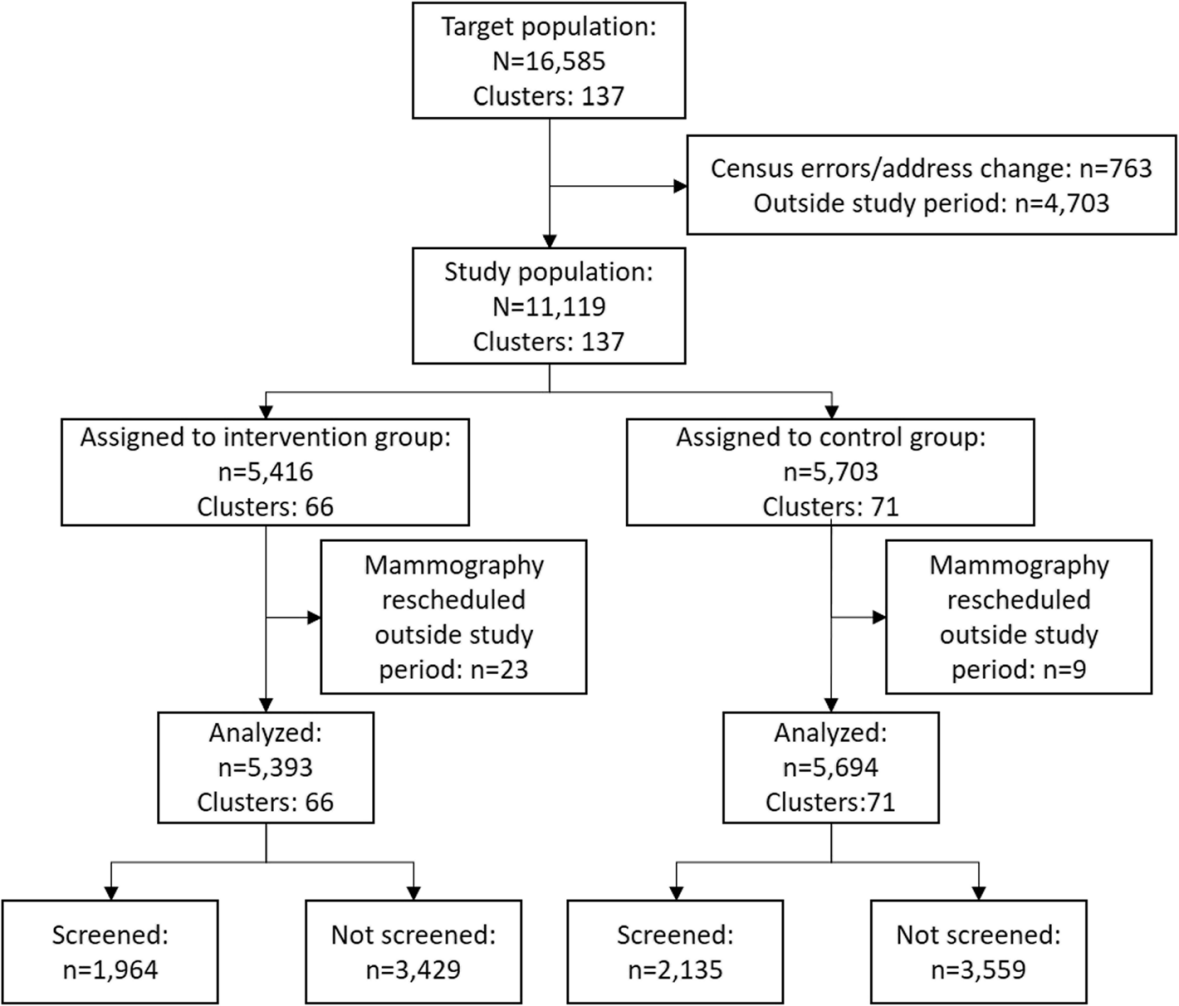
Flow diagram of the trial

A total of 11,119 women were included in this trial in 137 clusters, 5416 were assigned to the intervention group (66 clusters) and 5703 to the control group (71 clusters) (Fig. 1). The baseline characteristics of the two groups were similar, except that there was a larger number of women from Hospital B than Hospital A in the intervention group. There was also a slightly higher percentage of women born in Asia and Oceania in the intervention group in Hospital A (Table 1).

**Table 1:** Characteristics of the women included in the study. Intention-to-treat analysis

	Hospital A				Hospital B				Total			
	Intervention Group		Control Group		Intervention Group		Control Group		Intervention Group		Control Group	
	n	%	n	%	n	%	n	%	n	%	n	%
**Age** (median and IQR)	57	53–62	57	53–62	58	54–63	58	54–63	58	53–63	58	54–63
**Place of birth**												
Spain	1460	56.8%	1418	56.2%	1956	69.3%	2203	69.5%	3416	63.3%	3621	63.6%
Europe or North America	143	5.6%	174	6.9%	108	3.8%	107	3.4%	251	4.7%	281	4.9%
Center or South America	301	11.7%	292	11.6%	220	7.8%	261	8.2%	521	9.7%	553	9.7%
Asia or Oceania	515	20.0%	448	17.8%	33	1.2%	42	1.3%	548	10.2%	490	8.6%
Africa	96	3.7%	150	5.9%	12	0.4%	14	0.4%	108	2.0%	164	2.9%
*Missing*	55	2.1%	40	1.6%	494	17.5%	545	17.2%	549	10.2%	585	10.3%
**Study level**												
Primary or less	633	24.6%	667	26.4%	118	4.2%	144	4.5%	751	13.9%	811	14.2%
High-school or PT	1098	42.7%	1046	41.5%	1543	54.7%	1767	55.7%	2641	49.0%	2813	49.4%
Higher level	398	15.5%	419	16.6%	997	35.3%	1066	33.6%	1395	25.9%	1485	26.1%
*Missing*	441	17.2%	390	15.5%	165	5.8%	195	6.1%	606	11.2%	585	10.3%
**Initial or subsequent screening**												
Initial screening	1188	46.2%	1151	45.6%	2101	74.4%	2306	72.7%	3289	61.0%	3457	60.7%
Subsequent screening	1381	53.7%	1371	54.4%	722	25.6%	866	27.3%	2103	39.0%	2237	39.3%
*Missing*	1	0.0%	0	0.0%	0	0.0%	0	0.0%	1	0.0%	0	0.0%
**Total**	2570	100%	2522	100%	2823	100%	3172	100%	5393	100%	5694	100%

*PT* Professional Training.

**Fig. 2:**
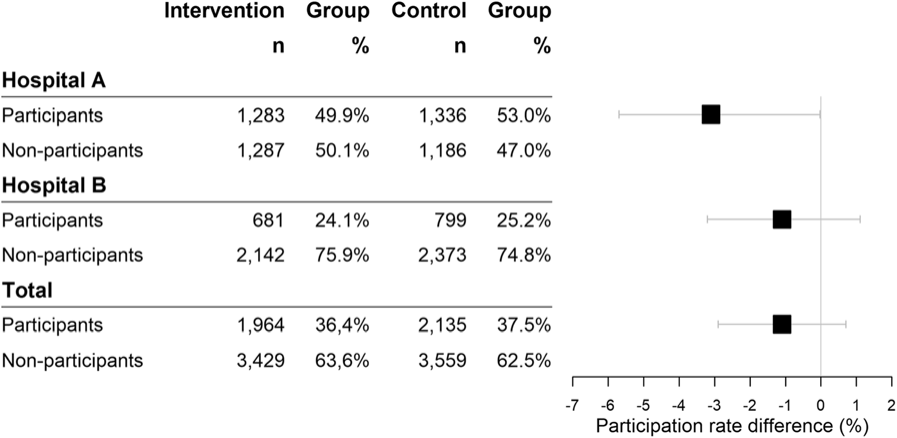
Participation in the breast cancer screening program. Intention-to-treat analysis

Overall, there were no differences in participation between the intervention and the control groups. A total of 36.4% (1964/5393) of the women in the intervention group participated in the breast cancer screening program versus 37.5% in the control group (2135/5694). The difference in participation of - 1.1% was not statistically significant (95% CI: − 2.9 to 0.7%, *p*-value = 0.240). However, in the analysis stratified by hospital, participation was lower in Hospital A in the intervention group (49.9%, 1283/2570) than in the control group (53%, 1336/2522) with a statistically significant difference of − 1.4% (95% CI, − 5.7% to − 0.03%, *p*-value = 0.029). In Hospital B, participation was 24.1% in the intervention group (681/2823) and 25.2% in the control group (799/3172), with no differences between groups in this hospital (*p*-value = 0.339) (Fig. 2).

Non-participants were mostly from Hospital B (62.5% of the intervention group and 66.7% of the control group) and most of them had never participated in our screening program (85.1% of the intervention group and 85.6% of the control group) (Supplementary Table 2).

**Fig. 3:**
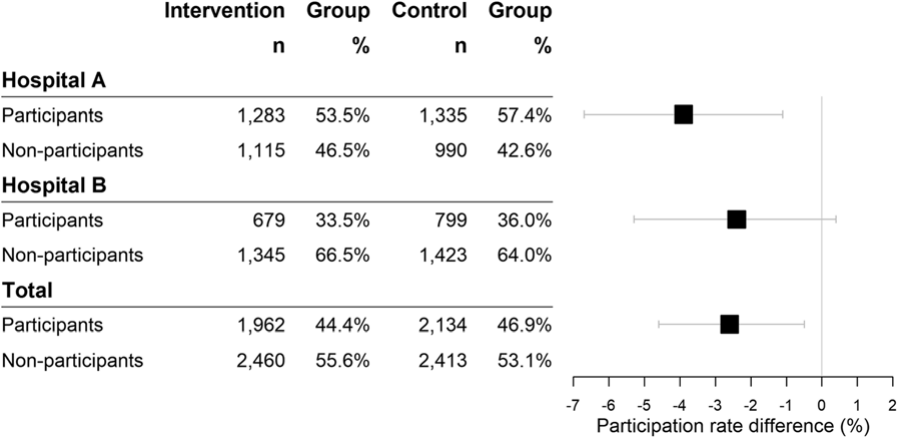
Participation in the breast cancer screening program. Per-protocol analysis

In the per-protocol analysis, participation was lower in the intervention group than in the control group (− 2.6, 95% CI: − 4.6% to − 0.5%, *p*-value = 0.015). In the stratified analysis, participation was lower in both hospitals, and was almost four percentual points lower in Hospital A (− 3.9, 95% CI: − 6.7% to − 1.1%, *p*-value = 0.007), but the difference was not statistically significant in Hospital B (*p-*value = 0.099) (Fig. 3). In the per-protocol analysis, excluded women were mostly from Hospital B (82.3% of the intervention group and 82.8% of the control group) and most had never participated in our screening program (89.5% of the intervention group and 90.0% of the control group) (Supplementary Table 3).

## Discussion

### Main findings

In our study, an information leaflet on the risk-benefit balance of breast screening did not appear to influence overall participation in the program. However, in one hospital attending a population with lower socioeconomic status the leaflet reduced participation (− 1.4% in the intervention group in Hospital A). Furthermore, the per-protocol analysis showed an overall lower participation in the intervention group (− 2.6%) in both hospitals (− 3.9% in Hospital A and − 2.4% in Hospital B), although this difference was not statistically significant in Hospital B.

### Comparison with previous studies

We found that providing explicit information about the risk-benefit balance of screening for the first time to women may not dramatically affect participation in the program. However, this intervention reduced participation in areas with a low socioeconomic status. Similar to our overall results, a randomized controlled study in Italy found no differences in participation among women who received extensive information material [15]. That study evaluated the impact of a comprehensive leaflet with additional information, which explained the balance in greater depth than our leaflet. Our results are also consistent with those of a German study reporting that providing more information about screening did not seem to affect women’s intention to participate in breast cancer screening [16]. Conversely, several other studies have suggested that providing information on the benefits and adverse effects of breast cancer screening decreases women’s intention to be screened [11, 12, 14, 17]. In agreement with these studies, the participation rate decreased in one of our hospitals.

Of note, how the information is presented may influence its effect. One of the studies was criticized because the decision aid placed too much weight on informing women of the risks of screening [18]. We believe that our leaflet is well-balanced and it clearly shows that the Catalonian Health Department invites women to be screened. However, the presentation of the leaflet could partly explain the differences in participation found in one of the centres. Overall, we did not find that providing information increased attendance in any group, or in the published literature.

A systematic review found that the use of decision aids made younger women (those around the age at which women begin to receive screening invitations) reluctant to participate in breast cancer screening programs [13]. In our trial, we found no differences in age between the intervention and control groups. Moreover, another study found an inverse relationship between age and reading information on breast cancer screening, a finding that contradicts the possible explanation of a lower participation of young women in Hospital A due to the leaflet [19]. On the other hand, another study argued that it was unlikely that women who have never participated in screening read screening information [19]. In Hospital B, most women had never participated in our screening program and so there is a possibility that a lower willingness to read screening information in this group could explain the absence of an effect of the leaflet in that hospital. However, we were unable to assess this possibility as we did not have a measure of reading the leaflet.

Women who have never participated in screening have been found to assign more weight to adverse effects than women who have participated at some point in the screening program [9, 20]. In our study, there was a similar proportion of these women in the intervention and control groups, and therefore this tendency seems unlikely to have influenced our results. However, another factor that has been related to a higher perceived importance of screening harms and lower perceived benefits is awareness of recent breast screening recommendations [10]. This hypothesis may be reflected in our results: women in Hospital B could be less willing to read the leaflet than those in Hospital A; in the intervention groups, attendance was unaffected in Hospital B but was reduced in Hospital A.

In a study focusing solely on non-participant women in Scotland, 55.5% of them had a history of non-attendance at screening [21]. In our study, around 85% of non-participant women had never attended our screening program. A possible explanation for our results could be that the areas included in our study have a high proportion of women who are disengaged from our screening program, and thus are not influenced by the information that we provide.

Another possibility could be that people do not usually read the information provided in the invitation letter, and decide to participate in the screening program based on information known beforehand through general practitioners, family or other sources of information.

The participation rate in this study was lower than that for the whole screening program in the last decade of around 55%. The low participation rate in Hospital B is probably explained by the high uptake of private breast screening in the area covered by this hospital. In addition, the COVID-19 pandemic started at the beginning of March 2020. Although this was the end of the study period, it could have negatively affected the expected participation to some extent.

### Strengths and limitations

The main strength of our study is the randomized controlled design, with information about women’s actual participation in the breast cancer screening program. This allowed us to assess whether providing information on the benefits and adverse effects of screening along with the invitation to the program affects participation.

Our trial also has several limitations. One of them is that we randomized letter processing days rather than individual women. However, processing days are similar to natural homogeneous grouping of the population, and within each day the population is heterogeneous, so we do not consider that this influenced the internal validity of the study. Another limitation is that the catchment areas included may not be representative of the general population, and thus the results may not be applicable to other populations, as they include areas with high socioeconomic status and others with low socioeconomic status. For that reason, we conducted an analysis stratified by hospital. Consequently, the stratified analysis may not have sufficient statistical power to detect statistically significant differences in Hospital B.

The follow-up was initially established at 90 days after the scheduled date of the mammogram, but this period overlapped with the SARS-CoV-2 pandemic, during which time mammogram appointments were cancelled. Thus, once the trial had started, we were forced to limit the follow-up to 30 days. However, around 90% of the women who were actually screened did so before that period, so we believe that shortening the follow-up probably did not alter the results of the study. In addition, we retrospectively registered the trial. However, we explained throughout the manuscript all deviations from the protocol study.

## Conclusions

Although the new Catalan information leaflet on the risk-benefit balance of mammography screening did not appear to significantly affect screening attendance, participation was lower in the area with low socioeconomic status. Screening programs sending material with detailed information on the benefits and adverse effects of breast cancer screening should closely monitor participation, particularly in areas with low socioeconomic status. There is a need for further research to assess the factors associated with non-attendance and their association with information leaflets.

## Supplementary Information

**Additional file 1.**

**Additional file 2.**

## Data Availability

The datasets used and/or analysed during the current study are available from the corresponding author on reasonable request.
