# Supplementary material for: Effect of an information leaflet on breast cancer screening participation: A cluster randomized controlled trial
Source: BMC Public Health. 2021 Jul 3;21:1301. doi: 10.1186/s12889-021-11360-0 (PMC8254065; doi:10.1186/s12889-021-11360-0)
Supplement: Supplementary file 1 — Additional file 1. [file 12889_2021_11360_MOESM1_ESM.pdf]

BREAST CANCER EARLY DETECTION

Breast cancer is the most common cancer in women in Catalonia.

Each year, 3,400 cases are diagnosed in women aged 50 or older. Most survive but 850 women die from the disease each year.

Early detection, also known as mammography screening, allows **breast cancer to be detected at a very early stage**, when it has not yet been noticed and does not produce symptoms, and when **the possibility of cure is highest**.

The Department of Health invites women aged 50 to 69 years to have a free mammogram every two years.

In most cases, early detection is beneficial but, because it can have adverse effects, women take the final decision to have a mammogram or not. Health professionals are on hand to advice and guide women when making this decision.

The information in this leaflet aims to help women decide on their participation in the breast cancer screening program of the Department of Health

WHAT IS A MAMMOGRAM?

Mammography is the most effective test for detecting breast cancer in the very early stages.

Mammography is an **x-ray of the breast**. Two x-rays are taken of each breast: one from above and the other from the side.

To get the clearest image, the breast must be squeezed against the machine. Some women may feel pain but it disappears quickly.

The procedure takes between 5 and 10 minutes.

You will learn **the results of the mammogram either by telephone or by mail in a few weeks**.

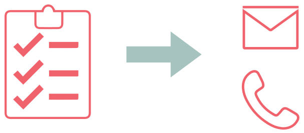

In some women (**60 out of 1,000 women** who have a mammogram) the results are unclear. These women undergo **more tests** to confirm or rule out possible cancer.

In the vast majority of cases, these tests rule out the presence of cancer. Only 5 out of these 60 women will be diagnosed with cancer.

Mammography does not prevent women from getting cancer.

WHAT ARE THE BENEFITS OF EARLY DETECTION?

Screening reduces the risk of dying from breast cancer.

For every 1,000 women who regularly have a mammogram every 2 years from the age of 50 to 69 years, 5 deaths from breast cancer are avoided.

Screening allows less aggressive treatments with fewer side effects if cancer is detected in an initial stage.

WHAT ARE THE ADVERSE EFFECTS OF SCREENING?

Some cancers can be detected that would never have threatened the lives of affected women.

Some cancers grow so slowly that they would never have posed a risk to life.

However, because it is still not known which cancers will progress and which wont, all cancers must be treated. This may lead to unnecessary treatments and side effects. This is known as overdiagnosis and overtreatment.

For every 1,000 women who have a mammogram every 2 years from the age of 50 to 69, between 5 and 10 will be diagnosed with some type of breast cancer or premalignant lesions that would never have become life-threatening.

In some women the results of the mammogram may be unclear and more tests will be necessary.

In most cases, cancer will be ruled out. These “false alarms” when no cancer is detected, can cause women to worry.

There is also the possibility of not detecting a cancer that has developed.

Consequently, if you notice any alterations in your breasts, it is important to visit your doctor.

Breast cancer early detection program

Each year, the Department of Health assesses the results of the Program and carries out quality control to enhance the programs quality and guarantee that the benefits of having a mammogram outweigh the undesired effects.

You can find a version of this leaflet in Spanish at the following link:

www.aspb.cat/cancer/mama

More information can be found on early detection of breast cancer and on the program:

061 CatSalut Respon

Breast cancer early detection program

Breast cancer

Mammographic early detection

Generalitat de Catalunya
Departament de Salut
