# Supplementary material for: Effect of an information leaflet on breast cancer screening participation: A cluster randomized controlled trial
Source: BMC Public Health. 2021 Jul 3;21:1301. doi: 10.1186/s12889-021-11360-0 (PMC8254065; doi:10.1186/s12889-021-11360-0)
Supplement: Supplementary file 2 — Additional file 2. [file 12889_2021_11360_MOESM2_ESM.docx]

**Supplementary Table 1.** Characteristics of the women included in the study. Per-protocol analysis.

|  | **Hospital A** | | | | **Hospital B** | | | | **Total** | | | |
| --- | --- | --- | --- | --- | --- | --- | --- | --- | --- | --- | --- | --- |
|  | **Intervention Group** | | **Control Group** | | **Intervention Group** | | **Control Group** | | **Intervention Group** | | **Control Group** | |
|  | n | % | n | % | n | % | n | % | n | % | n | % |
| **Age** (median and IQR) | 57 | 53-62 | 57 | 53-62 | 58 | 54-63 | 58 | 54-63 | 58 | 53-63 | 58 | 54-63 |
|  |  |  |  |  |  |  |  |  |  |  |  |  |
| **Place of birth** |  |  |  |  |  |  |  |  |  |  |  |  |
| Spain | 1,316 | 54.9% | 1,253 | 53.9% | 1,339 | 66.2% | 1,479 | 66.6% | 2,655 | 60.0% | 2,732 | 60.1% |
| Europe or North America | 133 | 5.5% | 162 | 7.0% | 87 | 4.3% | 93 | 4.2% | 220 | 5.0% | 255 | 5.6% |
| Central or South America | 293 | 12.2% | 277 | 11.9% | 202 | 10.0% | 234 | 10.5% | 495 | 11.2% | 511 | 11.2% |
| Asia or Oceania | 511 | 21.3% | 446 | 19.2% | 32 | 1.6% | 40 | 1.8% | 543 | 12.3% | 486 | 10.7% |
| Africa | 94 | 3.9% | 149 | 6.4% | 10 | 0.5% | 12 | 0.5% | 104 | 2.4% | 161 | 3.5% |
| *Missing* | 51 | 2.1% | 38 | 1.6% | 354 | 17.5% | 364 | 16.4% | 405 | 9.2% | 402 | 8.8% |
|  |  |  |  |  |  |  |  |  |  |  |  |  |
| **Educational level** |  |  |  |  |  |  |  |  |  |  |  |  |
| Primary or less | 610 | 25.4% | 653 | 28.1% | 106 | 5.2% | 120 | 5.4% | 716 | 16.2% | 773 | 17.0% |
| High-school or PT | 999 | 41.7% | 932 | 40.1% | 1,061 | 52.4% | 1,201 | 54.1% | 2,060 | 46.6% | 2,133 | 46.9% |
| Higher education | 363 | 15.1% | 364 | 15.7% | 717 | 35.4% | 747 | 33.6% | 1,080 | 24.4% | 1,111 | 24.4% |
| *Missing* | 426 | 17.8% | 376 | 16.2% | 140 | 6.9% | 154 | 6.9% | 566 | 12.8% | 530 | 11.7% |
|  |  |  |  |  |  |  |  |  |  |  |  |  |
| **Initial or subsequent screening** | |  |  |  |  |  |  |  |  |  |  |  |
| Initial screening | 1,049 | 43.7% | 985 | 42.4% | 1,371 | 67.7% | 1,440 | 64.8% | 2,420 | 54.7% | 2,425 | 53.3% |
| Subsequent screening | 1,349 | 56.3% | 1,340 | 57.6% | 653 | 32.3% | 782 | 35.2% | 2,002 | 45.3% | 2,122 | 46.7% |
| **Total** | 2,398 | 100% | 2,325 | 100% | 2,024 | 100% | 2,222 | 100% | 4,422 | 100% | 4,547 | 100% |

PT: Professional Training

**Supplementary Table 2.** Characteristics of non-participant women. Intention-to-treat analysis.

|  | **Non participants** | | | | **Participants** | | | |
| --- | --- | --- | --- | --- | --- | --- | --- | --- |
|  | **Intervention Group** | | **Control Group** | | **Intervention Group** | | **Control Group** | |
|  | n | % | n | % | n | % | n | % |
| **Age** (median and IQR) | 57 | 53-62 | 57 | 53-62 | 59 | 54-63 | 59 | 54-64 |
|  |  |  |  |  |  |  |  |  |
| **Place of birth** |  |  |  |  |  |  |  |  |
| Spain | 2,084 | 60.8% | 2,172 | 61.0% | 1,332 | 67.8% | 1,449 | 67.9% |
| Europe or North America | 163 | 4.8% | 180 | 5.1% | 88 | 4.5% | 101 | 4.7% |
| Center or South America | 303 | 8.8% | 297 | 8.3% | 218 | 11.1% | 256 | 12.0% |
| Asia or Oceania | 339 | 9.9% | 299 | 8.4% | 209 | 10.6% | 191 | 8.9% |
| Africa | 49 | 1.4% | 72 | 2.0% | 59 | 3.0% | 92 | 4.3% |
| *Missing* | 491 | 14.3% | 539 | 15.1% | 58 | 3.0% | 46 | 2.2% |
|  |  |  |  |  |  |  |  |  |
| **Study level** |  |  |  |  |  |  |  |  |
| Primary or less | 434 | 12.7% | 427 | 12.0% | 317 | 16.1% | 384 | 18.0% |
| High-school or PT | 1,904 | 55.5% | 1,952 | 54.8% | 737 | 37.5% | 861 | 40.3% |
| Higher level | 839 | 24.5% | 929 | 26.1% | 556 | 28.3% | 556 | 26.0% |
| *Missing* | 252 | 7.3% | 251 | 7.1% | 354 | 18.0% | 334 | 15.6% |
|  |  |  |  |  |  |  |  |  |
| **Center** |  |  |  |  |  |  |  |  |
| Hospital A | 1,287 | 37.5% | 1,186 | 33.3% | 1,283 | 65.3% | 1,336 | 62.6% |
| Hospital B | 2,142 | 62.5% | 2,373 | 66.7% | 681 | 34.7% | 799 | 37.4% |
|  |  |  |  |  |  |  |  |  |
| **Initial or subsequent screening** | |  |  |  |  |  |  |  |
| Initial screening | 2,919 | 85.1% | 3,046 | 85.6% | 370 | 18.8% | 411 | 19.3% |
| Subsequent screening | 509 | 14.8% | 513 | 14.4% | 1,594 | 81.2% | 1,724 | 80.7% |
| *Missing* | 1 | 0.0% | 0 | 0.0% | 0 | 0.0% | 0 | 0.0% |
|  |  |  |  |  |  |  |  |  |
| **Total** | 3,429 | 100% | 3,559 | 100% | 1,964 | 100% | 2,135 | 100% |

PT: Professional Training

**Supplementary table 3.** Characteristics of excluded women in the per-protocol analysis.

|  | **Women excluded in PP analysis** | | | | **Women included in PP analysis** | | | |
| --- | --- | --- | --- | --- | --- | --- | --- | --- |
|  | **Intervention Group** | | **Control Group** | | **Intervention Group** | | **Control Group** | |
|  | n | % | n | % | n | % | n | % |
| **Age** (median and IQR) | 59 | 54-64 | 58 | 54-63 | 58 | 53-63 | 58 | 54-63 |
|  |  |  |  |  |  |  |  |  |
| **Place of birth** |  |  |  |  |  |  |  |  |
| Spain | 761 | 78.4% | 889 | 77.5% | 2,655 | 60.0% | 2,732 | 60.1% |
| Europe or North America | 31 | 3.2% | 26 | 2.3% | 220 | 5.0% | 255 | 5.6% |
| Center or South America | 26 | 2.7% | 42 | 3.7% | 495 | 11.2% | 511 | 11.2% |
| Asia or Oceania | 5 | 0.5% | 4 | 0.3% | 543 | 12.3% | 486 | 10.7% |
| Africa | 4 | 0.4% | 3 | 0.3% | 104 | 2.4% | 161 | 3.5% |
| *Missing* | 144 | 14.8% | 183 | 16.0% | 405 | 9.2% | 402 | 8.8% |
|  |  |  |  |  |  |  |  |  |
| **Study level** |  |  |  |  |  |  |  |  |
| Primary or less | 35 | 3.6% | 38 | 3.3% | 716 | 16.2% | 773 | 17.0% |
| High-school or PT | 581 | 59.8% | 680 | 59.3% | 2,060 | 46.6% | 2,133 | 46.9% |
| Higher level | 315 | 32.4% | 374 | 32.6% | 1,080 | 24.4% | 1,111 | 24.4% |
| *Missing* | 40 | 4.1% | 55 | 4.8% | 566 | 12.8% | 530 | 11.7% |
|  |  |  |  |  |  |  |  |  |
| **Center** |  |  |  |  |  |  |  |  |
| Hospital A | 172 | 17.7% | 197 | 17.2% | 2,398 | 54.2% | 2,325 | 51.1% |
| Hospital B | 799 | 82.3% | 950 | 82.8% | 2,024 | 45.8% | 2,222 | 48.9% |
|  |  |  |  |  |  |  |  |  |
| **Initial or subsequent screening** | |  |  |  |  |  |  |  |
| Initial screening | 869 | 89.5% | 1,032 | 90.0% | 2,420 | 54.7% | 2,425 | 53.3% |
| Subsequent screening | 101 | 10.4% | 115 | 10.0% | 2,002 | 45.3% | 2,122 | 46.7% |
| *Missing* | 1 | 0.1% | 0 | 0.0% | 0 | 0.0% | 0 | 0.0% |
|  |  |  |  |  |  |  |  |  |
| **Total** | 971 | 100% | 1,147 | 100% | 4,422 | 100% | 4,547 | 100% |

PT: Professional Training
